# Mind the Age Gap: Expanding the Age Window for mRNA Vaccine Testing in Mice

**DOI:** 10.3390/vaccines13040370

**Published:** 2025-03-30

**Authors:** Muattaz Hussain, Agata Ferguson-Ugorenko, Rebecca Macfarlane, Natalie Orr, Samuel Clarke, Michael J. A. Wilkinson, Linda Horan, Yvonne Perrie

**Affiliations:** 1 Strathclyde Institute of Pharmacy and Biomedical Sciences, 161 Cathedral St., University of Strathclyde, Glasgow G4 0RE, UK; muattaz.y.hussain@strath.ac.uk (M.H.); agata.ugorenko@strath.ac.uk (A.F.-U.); rebecca.macfarlane.2017@uni.strath.ac.uk (R.M.); michael.wilkinson.100@strath.ac.uk (M.J.A.W.); l.horan@strath.ac.uk (L.H.); 2 Cytiva, 1055 Vernon Drive, Vancouver, BC V6A 3P4, Canada; natalie.orr@cytiva.com (N.O.); samuel.clarke@cytiva.com (S.C.)

**Keywords:** lipid nanoparticles, mRNA vaccines, murine models, pre-clinical, age-range

## Abstract

Background/Objectives: Murine models play a key role in guiding formulation and immunogenicity studies across various vaccine platforms, including mRNA-based vaccines. Typically, a narrow age range (6 to 8 weeks) is used in these studies. Here, we investigated whether widening this age range could provide greater flexibility in experimental design without impacting pre-clinical outcomes. Methods: To achieve this, we evaluated two commonly used lipid nanoparticle (LNP) formulations (based on SM102 and ALC-0315 ionizable lipids) containing either firefly luciferase or ovalbumin mRNA in female BALB/c mice aged 4, 8, and 16 weeks. LNPs were prepared and purified via microfluidics, and their size, polydispersity, zeta potential, and encapsulation efficiency were measured. Mice were injected intramuscularly, and the in vivo bioluminescence and antibody titers were measured to evaluate mRNA expression profiles and immunogenicity across the three age groups. Results: Our findings show that the 4-week-old mice exhibited higher protein expression following mRNA administration compared to the older groups; however, no significant differences were observed between the 8- and 16-week-old mice. Despite the initial higher protein expression, the antibody responses after the prime dose were lower in the 4-week-old mice compared to the other two groups. However, following the booster dose, antibody levels were comparable across all three age groups. Conclusions: By identifying a broader age range window, we provide greater flexibility in study design, enhance data comparability across studies, and promote more efficient use of animal resources, all while maintaining reliable and representative results in these murine models.

## 1. Introduction

Pre-clinical vaccine research relies heavily on animal models, with mice serving as a key model due to their well-characterized immune systems, genetic tractability, and relatively low cost [1]. Mouse models also play a critical role in guiding the formulation and design of lipid nanoparticles (LNPs) for mRNA vaccine delivery. Early-stage testing in mice helps assess protein expression profiles [2,3,4], immunogenicity [5], pharmacokinetics, and tissue distribution [6]. By administering various LNP compositions, including different lipid ratios [7], particle sizes [8], manufacturing conditions [9], and modifications to surface charge or targeting ligands [10], we can systematically evaluate how these variables affect both mRNA expression and immune responses. This iterative, data-driven approach not only accelerates the identification of promising formulations but also supports rational refinements to minimize off-target effects or inflammatory responses. By the time LNP candidates advance to larger animal models or early-phase clinical trials, they have typically undergone multiple rounds of pre-clinical validation in murine systems, significantly enhancing their likelihood of successful translation. However, as with all models, variability in outcomes can arise from factors such as mouse strain [11], biological sex [12], and age [13]. Despite their shortcomings, mice remain key to pre-clinical vaccine development. They permit large, standardized cohorts under controlled conditions, ensuring reproducibility and robust statistical power. Nevertheless, murine models require cautious interpretation given the inevitable differences between mice, larger pre-clinical models, and human immunobiology [14].

Previous studies have shown that immunological maturity and responsiveness in mice evolve continuously from weaning through adulthood. In response to this, many vaccine efficacy studies commonly use mice that are 6–8 weeks old. This age range is widely adopted because mice at this stage are considered young adults, with a relatively mature immune system and using mice within a specified window helps ensure more reliable and reproducible immune responses while minimizing the potential variability associated with developmental or age-related immune differences.

However, broadening the acceptable age range in murine studies can contribute to advancing the 3Rs (Replacement, Reduction, and Refinement) in pre-clinical research. Refinement emerges from minimizing the logistical challenges associated with strictly age-specific breeding cycles, as investigators gain greater leeway by selecting mice that are already available, rather than timing cohorts around a narrow window. Thus, expanding the commonly adopted age range may naturally reduce total animal use. Moreover, by aligning study designs with a proven age “safe zone” that yields consistent results, laboratories can decrease repetitive screening across narrowly confined age brackets. Consequently, demonstrating that moderately older or younger mice generate comparable LNP vaccine data both streamlines experimental workflows and supports a more efficient use of animals without compromising scientific quality.

In this paper, we investigate how mouse age affects mRNA expression and immune responses to mRNA vaccines formulated with two commonly used LNP formulations in a standard down-selection protocol. Mice were immunized at 4, 8, and 16 weeks. By comparing mRNA expression and antibody titers across these cohorts, we assessed whether age-based variability can influence the initial “go/no-go” decisions for LNP candidate selection, focusing on the age range in which differences in maturity have minimal impact on performance. Our objective was not to comprehensively characterize age-related immune responses in mice or to draw parallels with vaccine responses across human age groups, but rather to identify if we can safely broaden the age range used in mouse studies. This approach clarifies which deviations in mouse age do not significantly skew outcomes, allowing for greater flexibility in experimental design without compromising data comparability. By pinpointing a broader window where age-related factors are negligible, we aim to inform best practices in study planning, reduce variability, and use animal stocks more effectively while ensuring reliable and representative results.

## 2. Materials and Methods

### 2.1. Materials

The ionizable lipids SM-102 (8-[(2-hydroxyethyl)[6-oxo-6-(undecyloxy)hexyl]amino]-octanoic acid, 1-octylnonyl ester), ALC-0315 ([(4-hydroxybutyl)azanediyl]di(hexane-6,1-diyl) bis(2-hexyldecanoate)), and the PEGylated lipid ALC-0159 (methoxypolyethyleneglycoloxy(2000)-N,N-ditetradecylacetamide) were obtained from BroadPharm (San Diego, CA, USA). 1,2-distearoyl-sn-glycero-3-phosphocholine (DSPC) and 1,2-dimyristoyl-rac-glycero-3-methoxypolyethylene glycol-2000 (DMG-PEG2000) were purchased from Avanti Polar Lipids (Alabaster, AL, USA). Additional chemicals, such as cholesterol, citric acid, sodium citrate tribasic dehydrate, sulfuric acid, hydrogen peroxide (H_2_O_2_), Tween 20, and 3,3′,5,5′-tetramethylbenzidine dihydrochloride hydrate (TMB), were sourced from Merck Life Science (Hertfordshire, UK). mRNA constructs included EZ Cap™ Firefly Luciferase mRNA (5-moUTP) from Stratech Scientific (St Thomas’ Place, Cambridgeshire, UK) and 5-methoxyuridine (5moU)-modified ovalbumin-encoding mRNA (MRNA41) from OZ Bioscience (Marseille, France). For the in vivo protein expression studies, Vivo Glo luciferin substrate was procured from Promega Ltd. (Chilworth, UK). For immunoassays, Goat anti-Mouse IgG (H + L) HRP (A16066) was obtained from Invitrogen, while Goat Anti-Mouse IgG2a-HRP (1081-05) and Goat Anti-Mouse IgG1-HRP (1071-05) were from Southern Biotech (Birmingham, AL, USA). The Quant-it RiboGreen RNA assay kit was from Invitrogen (Waltham, MA, USA). All other solvents used were analytical grade and provided through in-house resources.

### 2.2. LNP Preparation

LNPs were produced using the NanoAssemblr™ Ignite™ nanoparticle formulation system from Cytiva (Vancouver, BC, Canada). The lipid formulation consisted of either DSPC:Cholesterol:SM-102:DMG-PEG2k at a molar ratio of 10:38.5:50:1.5% or DSPC:Cholesterol:ALC-0315:ALC-0159 at a ratio of 9.4:42.7:46.3:1.4% in ethanol (see Table 1), and the aqueous phase contained 0.11 mg/mL mRNA in a 50 mM citrate buffer at pH 4, resulting in an N/P ratio of 6 (ionizable lipid amine groups to mRNA phosphate groups). For our studies, mRNA encoding ovalbumin (OVA) was used as a model antigen in immunization experiments, and mRNA encoding Firefly luciferase (Fluc) served as the reporter gene in the expression assays. The microfluidic process was maintained at a 3:1 aqueous-to-organic flow ratio with a total flow rate of 20 mL/min. The resulting LNPs were purified using a Milipore Amicon^®^ Ultra-15 Centrifugal Filter Unit (100 kDa) from Merck Life Science (Hertfordshire, UK) after diluting the samples 40-fold in PBS (pH 7.4) and centrifuging at 2000× *g* at 4 °C until the desired volume was achieved.

### 2.3. LNP Characterization

The z-average hydrodynamic diameter, polydispersity index (PDI), and zeta potential were measured by dynamic light scattering using a Zetasizer Ultra (Malvern Panalytical Ltd., Worcestershire, UK), which uses a 633 nm laser and a detection angle of 173°. For particle size and PDI measurements, samples were diluted to a lipid concentration of 0.2 mg/mL in PBS. The zeta potential was determined after dilution to a final buffer concentration of 0.015 M PBS. The average particle size, PDI, and zeta potential are reported as the mean ± SD. The encapsulation efficiency was assessed using the RiboGreen assay. In brief, 50 µL of each sample was dispensed into a 96-well black plate under two conditions, with and without 0.1% (*w*/*v*) Triton X-100, to measure total mRNA and unencapsulated mRNA, respectively. After incubating the plate at 37 °C for 15 min to disrupt the LNPs, 100 µL of RiboGreen fluorescent dye was added, with dilutions of 200× for Triton-treated wells and 500× for untreated wells. Fluorescence was recorded on a GloMax^®^ Discover Microplate Reader (Promega Ltd., Chilworth, UK) at 480 nm excitation and 520 nm emission wavelengths. Encapsulation efficiency and mRNA recovery were then calculated using a standard curve generated from samples with and without Triton X-100.

### 2.4. In Vivo Expression and Antibody Responses

All animal procedures were conducted following the UK Home Office Animals Scientific Procedures Act of 1986 (UK project license PP1650440/personal license I52241434) and approved by an internal ethics board. Animals were housed in conventional MB1 polypropylene cages (NKP Isotec, East midlands, UK), with sawdust bedding (Eco-pure Premium, Datseand, Stockport, UK), nesting material (Sizzlenest, Datesand, Stockport, UK), wood chewblocks (Aspen bricks, Datesand, Stockport, UK), a clear plastic tube, and a rectangle red polypropelene mouse house (NKP Isotec, East midlands, UK). Tap water was provided ad libitum using clear plastic polycarbonate bottles (NKP Isotec, East midlands, UK) and a diet was also offered ad libitum using commercial extruded pellets (SDS RM1 Mouse Diet, Augy, France). Ambient humidity was kept between 45 and 65% and temperature between 19 and 23 degrees centigrade. Once a week, at cage change, mice were offered human-grade sunflower seeds and these were also offered as a treat (positive reinforcement) after any experimental procedures. The mice were free of major murine pathogens according to FELASA quarterly and annual health screening recommendations [15] (PCR positives only for *Helicobacter* spp., *Rodentibacter heylii* and *Entamoeba*). To examine the effect of age on mRNA expression, female BALB/cAnNCrl mice were randomized into groups of three mice per group using a random number generator and into three different age groups (4, 8, and 16 weeks). For the expression study, SM-102 or ALC-0315 benchmark LNPs loaded with Fluc mRNA were intramuscularly injected into both hind legs at a dose of 5 µg, after which the mice were anesthetized with isoflurane, administered a subcutaneous injection of d-luciferin (150 mg/kg), and imaged using an IVIS Spectrum (REVVITY (Wales, UK)) with Living Image software 4.8.2 at 15 min, 6 h, and 24 h post-injection; bioluminescence data were quantified as total flux within designated regions of interest. After each imaging session, each lasting around 5 min, mice were placed in their home cage under a heat lamp and, when fully recovered, were offered treats (sunflower seeds).

For immunization studies, female BALB/cAnNCrl mice were randomized into three groups (4 weeks, 8 weeks, and 16 weeks old) of 5 mice each using a random number generator. The mice received either SM-102 or ALC-0315 LNPs encapsulating OVA mRNA; the mice were primed intramuscularly with 5 µg on day 0, bled on day 27, boosted on day 28, and then euthanized on day 42 for final blood collection. Total IgG, IgG1, and IgG2a in serum were quantified by direct ELISA, where plates were coated overnight at 4 °C with 100 μL per well of 1 μg/mL OVA protein in 0.1 M carbonate buffer (pH 9.6), blocked with 10% FBS in PBS after washing, and incubated with diluted serum for 1 h at room temperature. Following further washes, horseradish peroxidase-conjugated goat anti-mouse antibodies (diluted 1:2500 for total IgG and 1:5000 for IgG1 and IgG2a) were added for another hour, and after a final wash, the reaction was developed using TMB substrate and terminated with 0.2 M H_2_SO_4_; absorbance was measured at 450 nm, and endpoint titers were determined based on the reciprocal of the final dilution yielding a positive signal.

### 2.5. Statistical Analysis

Data are presented as the mean values from independent experiments. Statistical analysis was carried out using GraphPad Prism 10.3.1, with ANOVA and corresponding post hoc tests applied as appropriate. A *p*-value below 0.05 was considered indicative of statistical significance.

## 3. Results

### 3.1. Assessing Age Selection Used in Published Pre-Clinical Mouse mRNA Vaccine Studies

**Figure 1:**
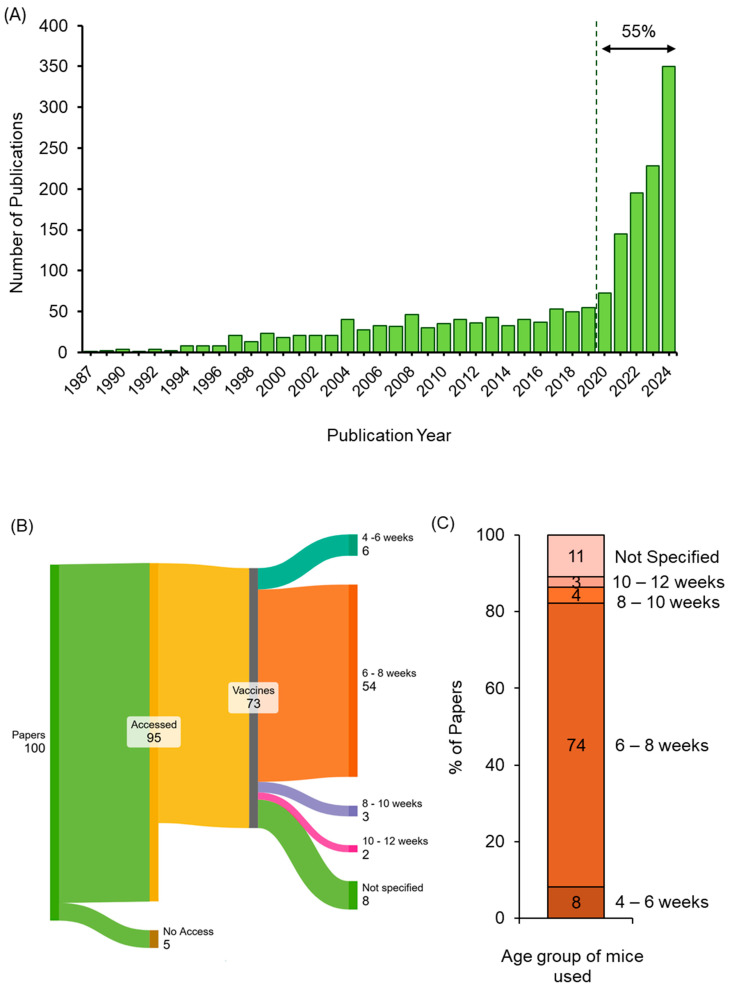
Overview of publication trends, study selection, and mouse age distribution in mRNA vaccine research. (**A**) Number of publications per year identified in PubMed using the search terms “mice, mRNA, vaccines”. The figure shows the trend in publications from 1987 to 31 January 2025 (access date: 31 January 2025). (**B**) A Sankey diagram illustrating the selection and categorisation of the most recent 100 publications from this PubMed search (generated by https://sankeymatic.com/build/; access date 31 January 2025). (**C**) Stacked column chart visualizing the percentage distribution of mouse age groups identified in the 73 relevant publications.

To investigate the age ranges commonly used in mRNA vaccine mouse studies, we conducted a review of recent publications. The search was performed on PubMed using the terms “mice, mRNA, vaccines” (accessed on 31 January 2025). Figure 1A illustrates the publication trend from 1987 to 2025, showing a marked increase in publications over time, particularly since 2020, which accounts for 55% of the total studies identified. This increase reflects the surge in pre-clinical mRNA vaccine research (in part driven by their adoption in the COVID-19 vaccines) and, in turn, a corresponding rise in murine models used in these investigations. Figure 1B displays a Sankey diagram illustrating the screening and categorisation process for the most recent 100 publications retrieved from the PubMed search. After excluding papers with restricted access (*n* = 5) and those not involving relevant mouse vaccine studies (*n* = 22), a total of 73 publications were identified. These studies were further analyzed to determine the reported ages of mice, which were grouped into five categories: 4 to 6 weeks, 6 to 8 weeks, 8 to 10 weeks, 10 to 12 weeks, and unspecified age. Figure 1C presents this distribution as a stacked column chart. Both Figure 1B,C demonstrate the predominantly narrow age range adopted across recent mRNA vaccine mouse studies, with the majority of publications (74%) reporting the use of 6- to 8-week-old mice. Based on these findings, we selected three age groups for further investigation: 4, 8, and 16 weeks.

### 3.2. LNP Formulation and Physicochemical Characterization

Two commonly used LNP formulations, based on SM-102 and ALC-0315 ionizable lipids, were selected for these studies due to their clinical relevance and established use in commercial COVID-19 vaccines. Both LNP formulations were prepared incorporating firefly luciferase (Fluc) mRNA, each employing a different ionizable lipid (SM102 versus ALC-0315) alongside DSPC, cholesterol, and a PEGylated lipid at predefined molar ratios (Table 1). Once manufactured, the LNPs were characterized to determine their particle size, polydispersity, zeta potential, mRNA encapsulation efficiency, and mRNA recovery (Table 2). In terms of their particle size, SM102 LNPs had an average particle size of 69 ± 1 nm, while ALC-0315 LNPs were larger at 89 ± 4 nm (Table 2). Despite this difference, both formulations had low PDI (PDIs < 0.1), indicating uniform particle size distributions (Table 2). The zeta potentials of both LNPs were also similar (−4 ± 3 mV for SM102 LNPs, −4 ± 1 mV for ALC-0315 LNPs), consistent with expected values for mRNA-LNPs. Both LNP formulations had similarly high mRNA encapsulation efficiencies (~92–93%) and comparable mRNA recoveries (~90–92%) (Table 2).

### 3.3. In Vivo mRNA Expression Across Age Groups

**Figure 2:**
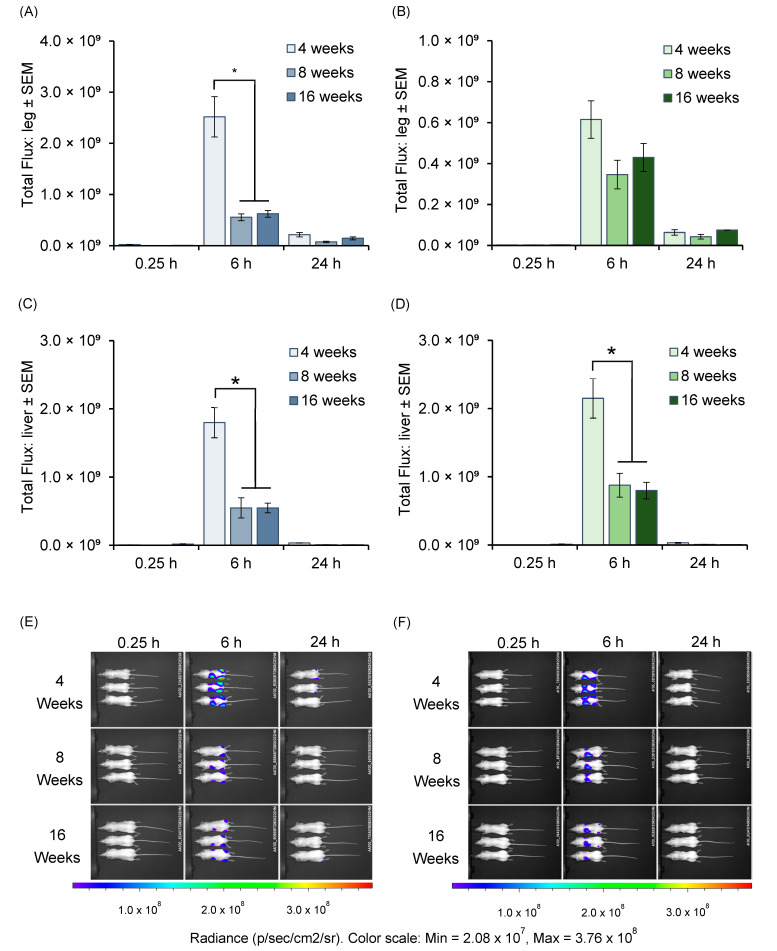
Bioluminescent imaging of firefly luciferase mRNA expression in mice of different ages. Mice were intramuscularly injected in both hind legs with mRNA (Fluc)-LNPs, and fluorescence intensity was measured in mice aged 4, 8 and 16 weeks old. The injected mRNA dose was 5 µg mRNA encapsulated in LNPs. Total flux from the hind leg region at 0.25, 6- and 24 h post-injection with SM102 LNPs (**A**) or ALC-0315 LNPs (**B**). Corresponding total flux from the liver region (**C**,**D**). Bars represent mean ± SEM from *n* = 3 mice per group (* *p* < 0.05). (**E**,**F**) Representative in vivo images illustrating luciferase expression at the same time points for SM102 LNPs (**E**) and ALC-0315 LNPs (**F**).

Following the characterization of SM102 and ALC-0315 LNPs, these formulations were then assessed for their in vivo mRNA (Fluc) expression in different age groups of mice by administering each formulation to mice aged 4, 8, and 16 weeks. Bioluminescence imaging was performed at 0.25, 6, and 24 h post-injection to assess the mRNA expression profiles (Figure 2) in mice that received SM102 LNPs (Figure 2A,C,E) or ALC-0315 LNPs (Figure 2B,D,F) encapsulating Fluc mRNA. The results show peak Fluc expression at 6 h post-injection in the injected hind legs (Figure 2A,B) and liver (Figure 2C,D). For SM102 LNPs (Figure 2A), 4-week-old mice exhibited the highest signal at 6 h, both at the injection site (Figure 2A) and liver (Figure 2C) (*p* < 0.05). In mice receiving ALC-0315 LNPs, there was no significant difference in mRNA expression at the injection site (Figure 2B). However, mRNA expression in the liver was again significantly (*p* < 0.05) higher in the livers of 4-week-old mice. Representative whole-animal images are shown in Figure 2E for SM102 and Figure 2F for ALC-0315 LNPs.

### 3.4. Antibody Responses in Different Age Groups of Mice Immunized with mRNA-LNPs

Having confirmed that both SM102 and ALC-0315 LNP formulations promoted mRNA expression in all the three different mouse ages, with higher expression observed in the younger age group, we next evaluated the humoral responses following immunization with ovalbumin (OVA)-encoding mRNA. By measuring serum antibody titers (total IgG, IgG1, and IgG2a), we aimed to determine whether the age-related differences seen in Fluc expression correlated with variations in vaccine-induced humoral immunity. Table 3 summarizes the physicochemical attributes of OVA-encoding mRNA LNPs. The results show that both the SM102 and ALC-0315 formulations had similar uniform particle sizes (z-average diameters of ~80–90 nm), low PDIs (<0.1), near-neutral zeta potential (−2 to −1 mV), and high mRNA encapsulation efficiencies (≥92%) and mRNA recoveries (≥91%) (Table 3).

**Figure 3:**
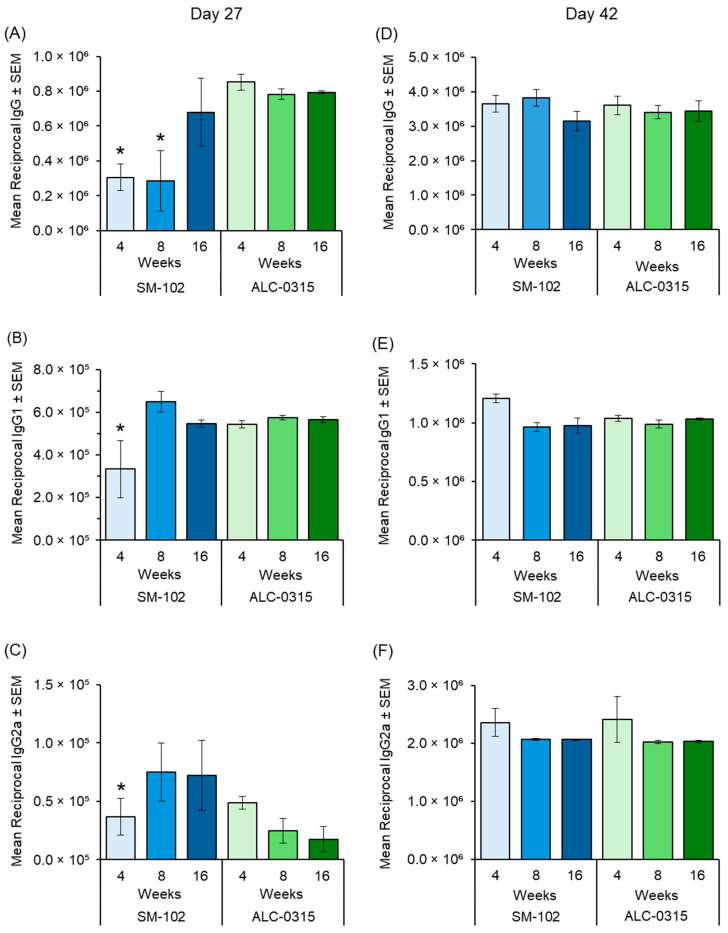
Serum Antibody responses in mice immunized with OVA-encoding mRNA LNPs. (**A**–**C**) Antibody titres on day 27 (post-prime), showing total IgG (**A**), IgG1 (**B**), and IgG2a (**C**) for SM102 LNPs (blue bars) and ALC-0315 LNPs (green bars) across 4-, 8-, and 16-week-old groups. (**D**–**F**) Corresponding titres on day 42 (two weeks post-booster). Bars represent mean reciprocal endpoint titres ± SEM (*n* = 5) (* *p* < 0.05).

Figure 3 shows the serum antibody responses against OVA-encoding mRNA delivered by SM102 LNPs (blue bars) or ALC-0315 LNPs (green bars) in mice immunized at 4, 8, or 16 weeks of age. Figure 3A–C show the antibody titres on day 27 (post-prime), whereas Figure 3D–F show the responses on day 42 (two weeks post-booster). Total IgG titres (Figure 3A) were significantly (*p* < 0.05) higher in 16-week-old mice compared to the 4- and 8-week-old cohorts when the mice received SM-102 LNPs, but this difference was not observed in mice receiving ALC-0315 LNPs. Similar profiles were observed for IgG1 (Figure 3B) and IgG2a (Figure 3C) with 4-week-old mice which received SM102 LNPs, again showing significantly lower titers (*p* < 0.05) compared to the 8- and 16-week-old cohorts, while no significant differences were found among age groups receiving ALC-0315 LNPs.

Following the booster dose, the total IgG levels (Figure 3D) were uniformly high across all ages for both SM102 and ALC-0315 LNP formulations (with no significant difference), effectively eliminating any early-age discrepancies observed at day 27. IgG1 (Figure 3E) and IgG2a (Figure 3F) levels similarly converged among the age groups, with no significant differences between the age groups or the two LNPs formulations (Figure 3). These findings suggest that initial age-related variations in antibody response were overcome by the second immunization. Overall, these data imply that while 4-week-old mice may produce lower antibody titres after a single dose with SM102 LNPs, booster immunization equalizes responses across the 4-, 8-, and 16-week-old cohorts.

## 4. Discussion

Before advancing to clinical trials, vaccines must undergo pre-clinical evaluations to ensure their safety and efficacy, with mice commonly being used due to their affordability, well-defined immune systems, and ease of genetic manipulation [16]. Indeed, numerous studies have used mice to evaluate immunogenicity, protective efficacy, and the down selection of candidate formulations for mRNA-based vaccines. For instance, ref. [17] presents a detailed protocol for designing and formulating mRNA-LNP formulations and outlines methods for analyzing immunization efficacy in mice.

Despite the central role of murine studies, other models (including hamsters, ferrets, and nonhuman primates) can offer broader insights into vaccine safety, efficacy, and immunogenicity, especially in contexts like COVID-19, and nonhuman primates, in particular, may provide closer parallels to human physiology [16]. However, mice remain pivotal for high-throughput investigations, enabling the rapid screening of multiple vaccine platforms under standardized conditions. To maximize the value of mouse models in preclinical research, it is crucial to understand their advantages, limitations, and variables (including age ranges) that may and may not impact on initial mRNA vaccine screening protocols. Obviously, age is only one among many factors potentially impacting on the host’s immune response to vaccination or to infection. Increased weight (obesity) and co-morbidities such as cardiometabolic conditions, are known to impair the immune system, increase the risk of severe infectious disease and lower vaccination efficacy in both humans and mice [18,19,20,21,22]. We (and others) have already shown the effect that biological sex can have on the immune response of BALB/c mice to immunization with mRNA vaccines, with females showing a much stronger IgG response when compared to males [12]. Hence, we chose to use metabolically/physiologically healthy mice with a fairly narrow weight range (not obese) and of the female sex, precisely to avoid potentially confounding factors whilst enhancing the probability of detecting a difference in terms of age.

**Figure 4:**
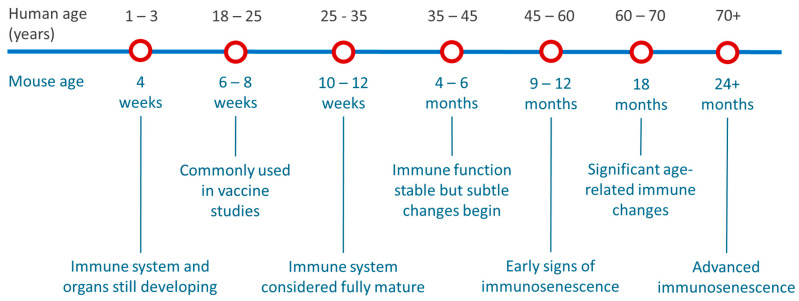
Age correlation between mice and humans. This figure illustrates the correlation between mouse age (in weeks and months) and the equivalent human age (in years). These values are approximate and depend on the specific physiological system being studied [23,24].

Figure 4 provides an approximate mapping of mouse age to human age. These estimates are based on typical laboratory mouse strains like BALB/c and C57BL/6. Immune system aging does not strictly follow chronological age, and functional assays can provide more accurate insights into age-related differences [23,24]. Figure 4 aligns with our findings from reviewing the most recent 100 publications with the 6 to 8 weeks age group being the most commonly adopted.

To consider the impact of mouse age in the testing of mRNA-LNP formulations, within our studies we used two clinically relevant LNP platforms and tested them using standard IVIS and vaccine protocols in mice of three different age cohorts. Our results show that the youngest mice show higher Fluc expression in the leg and liver and slightly reduced antibody responses after a single immunization. However, these differences were mitigated by a prime–boost regimen, resulting in comparable humoral immunity across all age groups. The differences observed in the mRNA expression profile, and the differences at the time of the prime vaccination may be attributed to the younger age (and lower weight) of the mice when they received their first dose. It has been shown, for example, that B cells in mice have an immature phenotype until they are 4 weeks old and that their T-cell responses mature at around 8 weeks of age [25,26,27]. However, by the time they received their booster, the youngest mouse group was 8 weeks old (an age commonly used in vaccine studies; Figure 4) and the oldest was 20 weeks old (immune system stable; Figure 4). Therefore, age-related effects were likely minimized, resulting in a more consistent immune response across the groups.

Although mRNA-LNP vaccine formulations have not previously been investigated in this context, numerous studies have shown that aging significantly impacts the murine immune system. For example, young and adult mice exhibit strong antibody responses to pneumococcal polysaccharide vaccines, while older mice display reduced and short-lived responses [28]. Similarly, universal influenza vaccine candidates induce more robust antigen-specific antibody and T-cell responses in young and middle-aged mice compared to elderly mice [29]. A live attenuated *Salmonella Typhimurium* vaccine also showed diminished immunogenicity in aged mice, with lower antibody titers and weakened T cell responses compared to adult mice [30]. Additionally, age-related decline in vaccine efficacy is observed in cancer vaccination, where DNA vaccines induce CD8 T-cell responses in young mice with metastatic breast cancer but fail to elicit similar responses in old mice [31].

In mice, one human year is almost equivalent to 9 mouse days when correlating their entire lifespan [32]. Immunosenescence becomes particularly pronounced in geriatric stages, characterized by reduced T cell proliferation, compromised germinal center formation, and aberrant inflammatory signaling [33]. Furthermore, recent studies on SARS-CoV-2 mRNA vaccines emphasize the importance of considering age in both clinical and pre-clinical research. An evaluation of the mRNA vaccine BNT162b2 using in vitro whole blood assays from adults (18–50 years) and older adults (≥60 year) demonstrated lower induction of Th-1 polarized cytokines and chemokines and these results were mapped to the results in aged (>10 months) mice in vivo [34]. The aged mice demonstrated impaired antibody induction at the three doses tested and waning immunity was more rapid compared with adult (6–12 weeks) mice [34]. The protective benefits of an mRNA booster dose across multiple age groups, including aged mice, was also shown, with the study reporting a significant decline in immune responses in 21-month-old mice 8 months after their primary vaccine. However, a booster dose substantially enhanced immune responses and was essential for protecting aged mice from severe Omicron infection.

Therefore, accounting for mouse age is not just an academic concern; it is crucial for ensuring the validity and reliability of pre-clinical vaccine assessments. However, using a broader age range for general vaccine screening studies supports the 3Rs (Replacement, Reduction, and Refinement) by increasing the choice of experimental subjects which, in turn, can reduce the number of animals needed and minimize stress factors associated with age-specific breeding cycles. In our studies, we have identified a broader age range (up to 16 weeks) that can be used in pre-clinical mRNA mouse studies, supporting the principles of the 3Rs (Replacement, Reduction, and Refinement). However, age-stratification in older mice can also provide valuable insights, as age-related immune responses can vary significantly. Incorporating these considerations into study designs will enhance the robustness of pre-clinical evaluations and support the ethical use of animals in research.

## 5. Conclusions

Pre-clinical studies play a critical role in vaccine development, offering essential insights into efficacy, safety, and mechanisms of action to guide vaccine formulation. Mice are the most commonly used animal model for such studies due to their well-characterized immune systems, low cost, and ease of handling. However, despite their widespread use, variability introduced by factors such as age has led to the adoption of overly cautious and narrow age ranges. This conservative approach can result in a suboptimal use of available animal stocks and limit the flexibility and efficiency of pre-clinical study designs. Nonetheless, age-related differences in immune system functionality, including changes in both innate and adaptive responses, can significantly influence vaccine efficacy and the associated immunological correlates. In our study, we demonstrate that 4-week-old mice exhibited higher initial mRNA protein expression compared to older cohorts, while no significant differences were observed between 8- and 16-week-old mice. Regarding antibody responses, early titers were lower in younger mice, but booster immunization equalized antibody levels across all age groups. Our findings indicate that the 8-to-16-week age range provides a similarly robust setting for evaluating mRNA-LNP vaccine performance, both in terms of protein expression and immunological outcomes. Consequently, these results support broadening the commonly used mouse age window beyond the standard 6–8 weeks, and increasing the age to 16 weeks, enabling greater flexibility in experimental design while maintaining reliability and validity in mRNA-based vaccine studies.

## Figures and Tables

**Table 1:** Composition of SM102 and ALC-0315 LNPs. Each formulation consists of DSPC, caniholesterol, an ionizable lipid, and a PEG lipid at the indicated molar percentages. SM102 LNPs contain SM102 as the ionizable lipid, whereas ALC-0315 LNPs use ALC-0315.

Lipid		SM102 LNPs	ALC-0315 LNPs
DSPC		10%	9.4%
Cholesterol		38.5%	42.7%
Ionizable lipid	SM102	50%	
	ALC-0315		46.3%
PEG lipid	DMG-PEG	1.5%	
	ALC-0159		1.6%

**Table 2:** Physico-chemical attributes of LNPs entrapping Fluc-mRNA. Results are expressed as the mean ± SD, *n* = 3.

EZ Cap™ Firefly Luciferase mRNA (5-moUTP)		
LNP Physico-Chemical Attributes	SM102 LNPs	ALC-0315 LNPs
z-average diameter	69 ± 1	89 ± 4
PDI	0.05 ± 0.01	0.02 ± 0.01
Zeta Potential (mV)	−4 ± 3	−4 ± 1
mRNA Encapsulation (%)	93 ± 1	92 ± 3
mRNA Recovery (%)	90 ± 8	92 ± 12

**Table 3:** Physico-chemical attributes of LNPs entrapping mRNA-encoding OVA physico-chemical attributes. Results are expressed as the mean ± SD, *n* = 6.

Ovalbumin-Encoding mRNA Modified with 5-Methoxyuridine (5moU)		
LNP Physicochemical Attributes	SM102	ALC-0315
z-average diameter	84 ± 11	87 ± 8
PDI	0.04 ± 0.02	0.06 ± 0.05
Zeta Potential (mV)	−2 ± 2	−1 ± 1
mRNA Encapsulation (%)	92 ± 1	95 ± 1
mRNA Recovery (%)	91 ± 8	97 ± 6

## Data Availability

The supporting dataset for this paper can be found at https://doi.org/10.15129/40a7fd59-8f87-47e3-aab6-ed86b7d7449b.
