# Correction: Hussain et al. Mind the Age Gap: Expanding the Age Window for mRNA Vaccine Testing in Mice. *Vaccines* 2025, *13*, 370

**DOI:** 10.3390/vaccines13090982

**Published:** 2025-09-19

**Authors:** Muattaz Hussain, Agata Ferguson-Ugorenko, Rebecca Macfarlane, Natalie Orr, Samuel Clarke, Michael J. A. Wilkinson, Linda Horan, Yvonne Perrie

**Affiliations:** 1 Strathclyde Institute of Pharmacy and Biomedical Sciences, 161 Cathedral St., University of Strathclyde, Glasgow G4 0RE, UK; 2 Cytiva, 1055 Vernon Drive, Vancouver, BC V6A 3P4, Canada

The authors would like to make the following corrections to the published paper [1]: In the originally published version of our article, References 1 and 14 were cited incorrectly. The corrected citations are provided below.

Corrected reference [2] to replace the original reference 1:

Kiros, T.G.; Levast, B.; Auray, G.; Strom, S.; van Kessel, J.; Gerdts, V. The Importance of Animal Models in the Development of Vaccines. In *Innovation in Vaccinology*; Baschieri, S., Ed.; Springer: Dordrecht, The Netherlands, 2012; pp. 251–264.

Corrected reference [3] to replace the original reference 14:

Carter, K.C.; Ata, D.T.; Aruleba, R.T.; Hurdayal, R. Preclinical Testing of Vaccine Candidates in Animal Models. In *System Vaccinology*; Prajapati, V., Ed.; Academic Press: Cambridge, MA, USA, 2022; pp. 257–280.

The authors state that the scientific conclusions are unaffected. These corrections were approved by the Academic Editor. The original publication has also been updated.
